# Case Report: Covid-19 in Multiple Sclerosis Patients Treated With Ocrelizumab: A Case Series

**DOI:** 10.3389/fneur.2021.691616

**Published:** 2021-10-20

**Authors:** Stefania F. De Mercanti, Marco Vercellino, Chiara Bosa, Anastasia Alteno, Valentina Schillaci, Marinella Clerico, Paola Cavalla

**Affiliations:** ^1^S.S.D. Patologie Neurologiche Specialistiche, AOU San Luigi Gonzaga, Orbassano, Italy; ^2^Department of Neurosciences and Mental Health, Multiple Sclerosis Center, AOU Città della Salute e della Scienza di Torino, Turin, Italy

**Keywords:** multiple sclerosis, ocrelizumab, COVID-19, SARS-CoV-2, disease-modifying treatment

## Abstract

**Introduction:** Limited data are available on the course of Coronavirus disease 2019 (COVID-19) in people with Multiple Sclerosis (MS). More real-world data are needed to help the MS community to manage MS treatment properly. In particular, it is important to understand the impact of immunosuppressive therapies used to treat MS on the outcome of COVID-19.

**Methods:** We retrospectively collected data on all confirmed cases of COVID-19 in MS patients treated with ocrelizumab, followed in two MS Centers based in University Hospitals in Northern Italy from February 2020 to June 2021.

**Results:** We identified 15 MS patients treated with ocrelizumab with confirmed COVID-19 (mean age, 50.47 ± 9.1 years; median EDSS, 3.0; range 1.0–7.0). Of these, 14 were confirmed by nasal swab and 1 was confirmed by a serological test. COVID-19 severity was mild to moderate in the majority of patients (*n* = 11, 73.3%; mean age, 49.73; median EDSS 3.0). Four patients (26.7%; mean age, 52.5 years; median EDSS, 6) had severe disease and were hospitalized; one of them died (age 50, EDSS 6.0, no other comorbidities). None of them had underlying respiratory comorbidities.

**Conclusion:** This case series highlights the large variability of the course of COVID-19 in ocrelizumab-treated MS patients. The challenges encountered by the healthcare system in the early phase of the COVID-19 pandemic might have contributed to the case fatality ratio observed in this series. Higher MS-related disability was associated with a more severe COVID-19 course.

## Introduction

Coronavirus disease 2019 (COVID-19) is a contagious respiratory disease caused by Severe Acute Respiratory Syndrome CoronaVirus 2 (SARS-CoV2), first identified in Wuhan, China in 2019, and responsible for the ongoing pandemic (1, 2). According to epidemiological data, about 80% of patients with COVID-19 develop a self-limiting illness, while 20% need hospitalization, and around 5% need ventilatory support (3, 4). In this last group, death occurs in about half of the cases (1, 5, 6). In the months following the pandemic outbreak, efforts have been made to identify risk factors associated with the worst outcomes: death has been related to older age and comorbidities such as cardiovascular and lung diseases, obesity, diabetes, and smoking habit (2, 7).

In this scenario, the management of people with MS (PwMS) has become more challenging: MS disease-modifying treatments (DMTs) may interfere with the immune system and increase the risk of infections (8, 9), with potential safety issues in the case of COVID-19 infection. As the pandemic situation is still very serious and widespread worldwide, more real-world data on COVID-19 in PwMS, particularly those receiving DMTs, are needed to manage MS treatment properly. In this case series, we will focus on one specific DMT, ocrelizumab. Ocrelizumab (Ocrevus®, Roche) is authorized in Europe for the treatment of adults with relapsing-remitting MS (RR-MS) and with early primary progressive MS (PP-MS) (10).

Ocrelizumab is a humanized monoclonal antibody that targets CD20 on B lymphocytes, with immunosuppressive effects due to peripheral B lymphocyte depletion (11). Ocrelizumab can also reduce T cells and has a slight effect on monocytes (12, 13). Infections occurring during ocrelizumab treatment can be serious (14, 15), due to persistent B cell depletion (14) and hypogammaglobulinemia (16). As of July 31, 2020, over 170,000 MS patients worldwide have been treated with ocrelizumab (17). Data on COVID-19 infection in these patients are controversial (17, 18). Spontaneous and detailed case reports may improve our knowledge of the impact of COVID-19 on ocrelizumab-treated patients.

Here, we describe a case series of confirmed COVID-19 infection in MS patients treated with ocrelizumab, in two MS Centers based in University Hospitals in Northern Italy, one of the first areas of Europe to experience the breakout of the COVID-19 pandemic at the beginning of 2020.

## Methods

We retrospectively collected data on all the cases of confirmed COVID-19 in ocrelizumab-treated MS patients, from February 2020 to June 2021, followed in two MS Centers based in University Hospitals in Northern Italy: (1) Multiple Sclerosis Center, Neurologia I U—AOU Città della Salute e della Scienza di Torino; (2) S.S.D. Patologie Neurologiche Specialistiche—AOU San Luigi Gonzaga. The total number of ocrelizumab-treated MS patients, as of June 2021, was 110 patients in Center 1 and 25 patients in Center 2.

All patients signed an informed consent for the data collection and publication in an anonymized form.

## Results

We identified 15 ocrelizumab-treated MS patients with confirmed COVID-19 (mean age, 50.47 ± 9.1 years; median EDSS 3.0, range 1.0–7.0). None of them was vaccinated before the infection since the majority of them contracted the disease before the vaccine was available. Anagraphic and anamnestic data on all the patients included in the case series are shown in Table 1.

**Table 1:** Anagraphic and anamnestic data.

**Case**	**Year of Birth**	**Age**	**Sex**	BMI^*^	**MS^°^ course**	**MS duration (year)**	**EDSS^£^**	**Comorbidities**	**Previous DMT^$^**	**Number of Ocrelizumab cycles before COVID-19 infection**	**Time between last Ocrelizumab cycle and COVID-19 symptoms (days)**	**White blood cells**	**Total lymphocytes**	**Ig^&^G**	**IgA**	**IgM**
1	1969	50	F	21.5	R	30	6	None	Teriflunomide, beta interferon 1a	3	36	7.26 × 1,000 uL	1.84 × 1,000 uL	NA	NA	NA
2	1980	40	M	25	R	6	1	Epilepsy	Teriflunomide, beta interferon 1a	2	73	4.42 10∧9/L	1.97 10∧9/L	879	168	55
3	1979	41	M	20.7	R	1	2	None	Natalizumab, Dimethylfumarate	1	10	10.23	3.69	751	89	159
4	1967	53	F	30.5	R	17	3.5	None	Dimethylfumarate, glatiramer acetate	4	11	5.84	1.2	968	353	207
5	1951	69	F	31.6	R	5	4	Hypertension, sick sinus syndrome	Teriflunomide	3	116	7.91	1.23	1,232	220	139
6	1970	49	F	19.1	R	1	2.5	Type 1-diabetes	None	1	27	5.72	1.3	1,264	335	45
7	1968	52	F	22.9	R	15	3	None	Beta interferone 1 a	3	137	7.05	2.1	868	147	145
8	1970	50	F	24.1	R	5	3	None	Natalizumab, teriflunomide	3	26	5.34	1.71	1,354	241	249
9	1968	52	F	19.5	R	1	2	None	None	3	136	5.5	1.2	1,403	250	107
10	1962	58	F	37.5	R	7	7	Graves' disease	Beta interferone 1 a	4	113	8.09	1.47	921	108	123
11	1959	62	M	23	R	36	5.5	Benign prostatic hyperplasia	Natalizumab, beta interferon 1 a	4	22	7.59	1.88	975	387	17
12	1980	41	F	24.5	R	12	4.5	None	Natalizumab, fingolimod	4	125	5.81	1.12	856	115	60
13	1967	53	F	17.2	R	16	5,5	HBV	Natalizumab, mitoxantrone, betaIFN1a	5	22	4.75	1.5	917	107	29
14	1968	54	M	27	R	1	4	Hypertension	None	1	48	5.94	1.1	1,083	97	74
15	1987	33	F	22.6	R	10	3	Hypertyroidism	Natalizumab, fingolimod, alemtuzumab	3	184	5.53	1.22	1,037	149	31
1	1969	50	F	21.5	R	30	6	None	Teriflunomide, beta interferon 1a	3	36	7.26 × 1,000 uL	1.84 × 1,000 uL	NA	NA	NA
2	1980	40	M	25	R	6	1	Epilepsy	Teriflunomide, beta interferon 1a	2	73	4.42 10∧9/L	1.97 10∧9/L	879	168	55
3	1979	41	M	20.7	R	1	2	None	Natalizumab, Dimethylfumarate	1	10	10.23	3.69	751	89	159
4	1967	53	F	30.5	R	17	3.5	None	Dimethylfumarate, glatiramer acetate	4	11	5.84	1.2	968	353	207

* *BMI, Body Mass Index*.

° *MS, Multiple Sclerosis*.

£ * EDSS, Expanded Disability Status Scale*.

$ *DMT, Disease Modifying Treatment*.

& * Ig, Immunoglobulin*.

*NA, not available*.

COVID-19 severity was mild to moderate in the majority of patients (*n* = 11, 73.3%; mean age 49.73; median EDSS 3.0). Four patients (26.7%; mean age 52.5 years; median EDSS 6) had severe disease and were hospitalized, and one of them died (age 50, EDSS 6.0, no other comorbidities). Three patients developed neurological symptoms during COVID-19 such as dizziness, worsening of pre-existing neurological symptoms, and increased leg stiffness. Eight patients had respiratory signs/symptoms (radiologically documented pneumonia, dyspnea, low blood oxygen saturation), but only three of them received oxygen therapy (one received mechanical ventilation, one received assisted non-invasive ventilation, and one received home oxygen therapy) (Table 2).

**Table 2:** Data about COVID-19.

**Case**	**Nasal swab positive/negative**	**Respiratory symptom**	**Delayed infusion Yes/NO**	**Days of delayed infusion**	**Fever Yes/No**	**COVID-19 treatment**	**Hospitalization Yes/NO**	**Antibodies against SARS-CoV2**	**Serological test type**	**Covid-19 outcome**	**COVID-19 sequelae**	**Vaccination Yes/No**
1	pos	Pneumonia			Y	Hydroxychloroquine, darunavir, ritonavir, ceftriaxone, steroids, nitric oxide, ozone, antitrombotic, NIV	Y	Not performed		Deceased		/
2	neg	Pneumonia	N		Y	Hydroxychloroquine, azithromycin, steroid	Y	IgM and IgG pos (27/5/2021)	ChLIA	Recovered		
3	pos (15/9/2020)		N		N	Paracetamol	N	IgG neg, IgM neg (18/5/2021)	ChLIA	Recovered		
4	pos (28/10/2020)		N		Y	Steroid	N	IgG pos, IgM neg /5/3/21)	ChLIA	Recovered	Increased fatigue	
5	pos (22/10/21)	SpO_2_ reduction	Y	30	N	Steroid, LMWH, azithromycin	N	IgM neg, IgG pos (20/4/2021), 12/20, 01/21, 02/21.	ChLIA	Recovered		Y
6	pos (7/11/20)		N		Y	/	N	Not performed		Recovered		Y
7	pos (23/10/20)		N		Y	Steroid therapy, amoxicilline	N	IgG neg (2/21)	ChLIA	Recovered		Y
8	pos (30/10/2020)		N		Y	Steroid therapy, amoxicilline	N	Not performed		Recovered		
9	pos (23/10/2020)		N		Y	/	N	Not performed		Recovered		
10	pos (9/12/20)	SpO_2_ reduction	Y	280	Y	Steroid therapy, antibiotic	Y	Not performed		Recovered		Y
11	pos (4/1/2021)	SpO_2_ reduction	N		Y	Steroid, heparin, antibiotic coverage, oxygen therapy	Y	Not performed		Recovered	Dyspnea, cycle of O_2_ therapy, worsening of neurological symtoms	Y
12	pos (14/2/2021)	Dyspnea, cough	Y	35	Y	Antibiotic therapy, steroid	N	IgG pos, IgM neg (1/6/2021)	ChLIA	Recovered	Cough, dyspnea, headache (improving)	
13	pos (20/3/21)	Dyspnea, SpO_2_ reduction (88%)	N		Y	Steroid, oxygen therapy	N	Not performed		Recovered		N
14	pos (2/4/2021)	Dyspnea	N		Y	Steroid therapy, antibiotic therapy	N	Not performed		Recovered	Ipogeusia, fatigue, increased legs stifness	N
15	pos (03/06/2021)		Y	38	Y	Heparin, antibiotic therapy	N	Not performed		Recovered		N
Case #	Nasal swab pos/neg	Type of symptom	Delayed infusion Y/N	Days of delayed infusion	Fever Y or N	Covid-19 treatment	Hospitalization	Antibodies against SARS-CoV2	Serological test type	Covid-19 outcome	Type of symptoms	vaccino Y/N
1	pos	Pneumonia			Y	Hydroxychloroquine, darunavir, ritonavir, ceftriaxone, steroids, nitric oxide, ozone, antitrombotic, NIV	y	Not performed		Deceased		/
2	neg	Pneumonia	N		Y	Hydroxychloroquine, azithromycin, steroid	Y	IgM and IgG pos (27/5/2021)	ChLIA	Recovered		
3	pos (15/9/2020)		N		N	Paracetamol	N	IgG neg, IgM neg (18/5/2021)	ChLIA	Recovered		

In nine patients, there was at least one risk factor associated with a more severe COVID-19 disease course: age > 50 years (*n* = 9); obesity (*n* = 3); hypertension (*n* = 2); diabetes (*n* = 1). None of them had underlying respiratory comorbidities; one patient had epilepsy since childhood and two patients had hyperthyroidism. The proportion of patients with an EDSS > 3 was 53.3%. In the last blood tests within 3 months before the onset of COVID-19, total leukocytes count and total lymphocyte counts were within normal limits in all patients. Likewise, serum Ig levels were within the normal range in all patients (for one patient this data was not available) (Table 1).

Comparing the patients with and without respiratory signs/symptoms during COVID-19 (radiologically-documented pneumonia, dyspnea, low blood oxygen saturation), the patients showing respiratory signs/symptoms showed a higher median EDSS than the patients without respiratory signs/symptoms (EDSS 5.0 vs. EDSS 3.0; Mann-Whitney *U*-test, *p* = 0.0150).

After COVID-19 recovery, six patients underwent a serological test to detect the presence of antibodies against SARS-CoV2 (quantitative test with chemiluminescent immunoassays): one patient tested positive for both IgM and IgG; three patients tested positive for IgG against SARS-CoV2; and two tested negatives (Table 2).

Four patients showed EDSS progression in the months following COVID-19 (Table 2). For two of them, this could be related to a delayed infusion and for one patient, this could be related to the prolonged hospitalization during and after COVID-19 (Table 2).

Over the following months, the majority of patients fully recovered. However, four patients were still symptomatic even after several months from COVID-19, presenting increased fatigue, worsening of neurological symptoms and, in one case, need for chronic oxygen therapy (Table 2).

Five patients received SARS-CoV2 vaccines based on mRNA technology, at least 3 months after COVID-19 recovery (Table 2).

### Case 1

A 50-year-old woman with a history of RR-MS since 1989 was treated with interferon beta 1a and teriflunomide before switching to ocrelizumab due to disease activity. On February 20, 2020, she underwent the most recent administration and her EDSS score was 6.0. On March 19, 2020, the patient presented to the emergency room (ER) with complaints of fever with syncope. Chest X-ray showed bronchopneumonia foci of inflammatory condensation, a swab for COVID-19 was carried out and, pending the outcome, the patient was discharged with amoxicillin-azithromycin antibiotic therapy. On March 26, due to the worsening of dyspnea, the patient came back to the ER with subsequent hospitalization. Chest X-ray showed multiple confluent parenchymal thickenings and the second swab for COVID-19 tested positive. Assisted non-invasive ventilation (NIV) was started, together with hydroxychloroquine, ceftriaxone, and antiviral therapy (darunavir and ritonavir). Due to the worsening of the general conditions and respiratory exchanges, on the second day after admission, the patient was transferred to the Intensive Care Unit (ICU) and underwent mechanical ventilation. After 4 days, for further worsening of respiratory exchanges, the patient received nitric oxide and steroid therapy. Because of increased platelets and hypertension, antithrombotic, and antihypertensive therapy with acetylsalicylic acid and analytics was added. A Chest CT scan showed bilateral pneumonia. The slow but progressive clinical and radiological improvement led on April 7 to suspend invasive ventilation and to start physiotherapy. On April 20, a new febrile episode occurred with negative blood cultures. Due to persistent anemia, the patient received a periodic blood transfusion. Bone marrow biopsy showed a hyporegenerative marrow, the absence of blasts, and the absence of viruses; in peripheral blood, no CD19+ lymphocytes were found at immunotyping. A Chest CT scan performed on May 13 showed a worsening of the pulmonary infiltrates on the left fields. The bronchoalveolar lavage showed persistence of positivity for SARS-CoV2. The patients had persistent hyperpyrexia up to 40°C; suspecting a persistence of COVID-induced interstitial pneumonia in a patient with a poor immune response, on May 18, an off-label treatment with ozone was carried out. This treatment was initially well-tolerated with an improvement of respiratory exchanges; however, 2 h after the end of the second ozone session, the patient died due to acute pulmonary edema with cardiorespiratory arrest.

### Casr 2

A 40-year-old male was diagnosed with RR-MS in 2014; epilepsy was also reported in his medical history. He was initially treated with beta-interferon and then switched to teriflunomide. In July 2019, therapy with ocrelizumab was started because of disease activity. EDSS score was 1.0. On April 18, 2020, he developed a fever and cough. He presented to the ER, a CT scan showed ground-glass opacity and interstitial abnormalities, and he was hospitalized. Chest X-ray showed inflammatory condensation. The patient underwent three nasal swabs for SARS-CoV2: all of them were negative, but the suspicion of COVID-19 pneumonia was high. He was treated with hydroxychloroquine, antimicrobial therapy, and steroids. On May 9, due to mild residual symptomatology, he was discharged and completed therapy at home. He completely recovered over the following days and, on May 12, a chest CT scan was normal. On May 27, he underwent a quantitative serological test that detected the presence of antibodies against SARS-CoV2, both immunoglobulin-M (IgM) and IgG (IgM 5.47 U/L and IgG 42.6 U/L).

### Case 3

A 41-year-old man was diagnosed with RR-MS in May 2019. He had no other medical illnesses and he started di-methyl-fumarate but switched to Natalizumab because of disease activity. In August 2020, a brain MRI showed disease activity and antibodies against Natalizumab tested positive. Therefore, Natalizumab was stopped and ocrelizumab was started in September 2020. After 10 days, he developed fatigue and nasal congestion. His cohabitant partner also developed similar symptoms and both of them tested positive by nasal swab for COVID-19. They were quarantined at home; the symptoms of the patient rapidly improved over the following days with no need for specific therapies. The patient tested negative by nasal swab on November 15. On May 18, 2021, he underwent a quantitative serological test that did not detect antibodies against SARS-CoV2.

### Case 4

A 53-year-old woman was diagnosed with RR-MS in 2003. She was treated with different DMTs, such as glatiramer acetate and dimethyl-fumarate, but she switched to ocrelizumab in February 2019 due to disease activity (fourth cycle on October 8, 2020). Her last EDSS score was 3.5 and she had no other serious medical illnesses. On October 19, 2020, she presented with vomit, diarrhea, headache, and fever. She tested positive by nasal swab for SARS-CoV2 on October 28. Due to mild symptomatology, she was self-quarantined at home, she was prescribed oral steroid therapy and gradually recovered over the following days. She tested negative by nasal swab on November 17, and she underwent a quantitative serological test in March 2021 that detected the presence of antibodies against SARS-CoV2.

### Case 5

A 69-year-old woman was diagnosed with RR-MS in 2015 and started teriflunomide in February 2016. Her comorbidities included hypertension and sick sinus syndrome (pacemaker wearer). Due to a persistent disease activity, she switched to ocrelizumab in March 2019. Her last EDSS score was 4.0.

On October 11, 2020, she developed dizziness, nausea, vomit, and fatigue, but no fever or cough. She underwent an urgent neurological visit, in suspicion of an MS relapse. Brain MRI was stable, and she tested positive for SARS-CoV2 by nasal swab on October 22. Steroid therapy, azithromycin, and low molecular weight heparin were started, and her symptoms remained stable over the following days while she was self-quarantined at home. She recovered in 2 weeks, but nasal swabs in the following month were still positive. She tested negative on January 20, 2021. One month later, she tested positive for IgG to SARS-CoV2 (detected with a quantitative test), and she also tested positive in the following test performed in April 2021.

### Case 6

A 49-year-old woman was diagnosed with aggressive RR-MS in June 2020, with a high lesion burden on MRI. Her EDSS was 2.5 and she suffered from type1-diabetes since childhood, treated with insulin therapy. She had a positive serology for the JC virus. After a careful discussion of the pro and cons of the available treatments, she started ocrelizumab on September 16, 2020. On October 29, 2020, she developed fever, cough, headache, and nasal congestion and tested positive for COVID-19 by nasal swab on November 7. Her symptoms improved over the following days without any specific treatment and were fully resolved.

### Case 7

A 52-year-old woman was diagnosed with RR-MS in October 2005. In 2019, she switched from beta-interferon to ocrelizumab (last cycle on June 8, 2020) because of evidence of disease activity. Her last EDSS score was 3.0 and she had no other medical illnesses. On October 23, 2020, she developed fever, cough, shortness of breath, anosmia, and ageusia. She started amoxicillin and prednisone following the advice of her doctor. Nasopharyngeal swab, performed on October 25, was positive for SARS-CoV2 and, due to mild symptoms, she was self-quarantined at home. She completely recovered over the following weeks except for a persisting anosmia and ageusia, and she tested negative by the nasal swab on November 16, 2020. In February 2021, she underwent a quantitative serological test that detected the presence of IgG against SARS-CoV2.

### Case 8

A 50-year-old woman was diagnosed with RR-MS in 2015. She was treated with teriflunomide and natalizumab before switching to ocrelizumab on September 16, 2019 due to the persistence of disease activity (third cycle on October 1, 2020). She had no other medical illness and her EDSS score was 3.0. On October 27, 2020, she reported fever, headache, nasal congestion, hyposmia, and ageusia. After a few days, a nasal swab confirmed the diagnosis of COVID-19. She started steroid therapy and gradually recovered without the need for hospitalization.

### Case 9

A 52-year-old woman was diagnosed with aggressive RR-MS in 2019 and was treated with ocrelizumab on May 23, 2019 (last cycle June 19, 2020). She had no other comorbidities and her EDSS score was 2.0. On October 23, 2020, she developed fever, fatigue, and bone pain. She rapidly recovered without therapy over the following days. Two nasal swabs were still positive for SARS-CoV2 (October 28 and November 11); she tested negative on January 27, 2021.

### Case 10

A 58-year-old woman was diagnosed with an active PP-MS in 2014. Her comorbidities included obesity and Graves' disease. Ocrelizumab therapy was started in September 2018. In June 2020, she was hospitalized with the suspicion of clinical and radiological relapse and treated with steroid therapy. Her EDSS score was 7.0. On August 18, 2020, she received the fourth cycle of ocrelizumab. On December 9, 2020, during a hospitalization in a rehabilitation facility, she developed cough, high fever, and vomit and tested positive for SARS-CoV2 by the nasal swab. She was immediately transferred to a dedicated ward and was treated with intravenous steroids and antibiotics for 3 weeks; she also needed oxygen support because of hypoxemia. She gradually and completely recovered over the following weeks and tested negative by nasal swab on January 20, 2021.

### Case 11

A 62-year-old man with a diagnosis of RR-MS was treated with several DMTs, such as beta-interferon and Natalizumab, before switching to ocrelizumab in April 2019. He received his last cycle on December 17, 2020. EDSS was 5.5 and he had no other medical illnesses except for benign prostatic hyperplasia. In December 2020, his cohabiting partner tested positive for SARS-CoV2 by nasal swab. For this reason, he performed various nasal swabs and, on January 4, 2021 he tested positive. He developed fever, nasal congestion, and cough on January 8, 2021. On January 20, due to the worsening of symptoms and the appearance of dyspnea, the patient was hospitalized. Steroids, low molecular weight heparin, and antibiotics (with three different therapeutic regimens) were used, and due to the worsening of respiratory exchanges, he received NIV. Over the following days his symptoms remained stable, he tested negative by nasal swab on February 4, 2021, and he was switched to oxygen therapy before being discharged on February 12, 2021. He gradually recovered over the following weeks and gradually tapered oxygen therapy at home.

### Case 12

A 40-year-old woman was diagnosed with RR-MS in 2008. She was treated with natalizumab but switched to fingolimod in 2013 due to a positive serology for the JC virus. Due to the persistent disease activity, she switched to ocrelizumab in February 2019 and she received her last cycle on September, 30, 2020. Her EDSS score was 4.5 and she had no other medical illnesses. On February 14, 2021, she developed fever (39.5°C), cough, headache, shortness of breath, nasal congestion, nausea, and lymph nodes enlargement. The next day, she tested positive for SARS-CoV2 by nasal swab. She gradually recovered over the following weeks, with a persistent cough, headache, and thoracic pain. After 2 months, she underwent a chest X-ray that showed thickened bronchovascular markings. She received steroid and antibiotic therapy, and she fully recovered over the following weeks. She received her fifth cycle of Ocrelizumab on May 5, 2021 and, on June 1, 2021, she underwent a serological test that detected the presence of antibodies (IgG) against SARS-CoV2.

### Case 13

A 53-year-old woman with a diagnosis of RR-MS was treated with several DMTs (beta-interferon, mitoxantrone, and natalizumab) before switching to ocrelizumab on February 13, 2019. She had no other medical illnesses (except for HBV positivity) and her EDSS score was 5.5. On March 22, 2021, 22 days after her fifth cycle of ocrelizumab, she developed fatigue, sore throat, headache, shortness of breath, and loss of taste, and a nasal swab tested positive for COVID-19. Over the following days, her oxygen blood saturation dropped below 90% and she received oxygen therapy for 10 days. She also received steroid therapy and paracetamol, fully recovered over the following days, and tested negative by nasal swab on April 16, 2021.

### Case 14

A 54-year-old man was diagnosed with an aggressive RR-MS in January 2021. He had no other medical illnesses, his EDSS score was 4.0, and he received his first cycle of ocrelizumab on January 28, 2021. On April 1, 2021 he developed leg stiffness, headache, fever (40°C), loss of taste and smell, and on the next day, he tested positive for COVID-19 by nasal swab. He received steroid and antibiotic therapy, but he did not fully recover despite testing negative on May 6, 2021. He still is symptomatic, with increased fatigue, persisting reduction of taste, and leg stiffness.

### Case 15

A 33-year-old woman with a 10-year history of MS disease was treated with several DMTs such as beta-interferon, natalizumab, fingolimod, and alemtuzumab, before switching to ocrelizumab on October 16, 2019. Her EDSS score was 3.0, and she suffered from hyperthyroidism. She received her last cycle on November 20, 2020 and 6 months later, few days before receiving the next cycle, she developed fever, cough, headache, and fatigue. She tested positive by nasal swab for COVID-19 on June 3, 2021, and she fully recovered over the following days with heparin and antibiotic therapy. She tested negative by nasal swab on June 19, and she received her fourth cycle of ocrelizumab on July 8, 2021.

## Discussion

Here, we describe an incident case series of 15 ocrelizumab-treated MS patients with confirmed COVID-19, followed at two MS Centers in Northern Italy, from the beginning of the pandemic in continental Europe in February 2020 to June 2021. Knowledge about the SARS-CoV2 virus itself and how it may affect MS patients is still limited (6, 18–20). The available data regarding COVID-19 in people with MS show that, in general, the MS population does not seem to be at a higher risk of death from COVID-19 (18–21). Major risk factors identified for a severe/fatal COVID-19 in the MS population are the same factors identified in the general population: older age (>50 years old), higher levels of disability, MS progressive course, and presence of comorbidities (6, 7, 18–20). The role of single DMTs on the COVID-19 disease course is not completely clear yet: mortality rates do not differ when considering specific DMT use (18, 19), but the frequency of severe COVID-19 could be higher in anti-CD-20 therapies compared to other DMTs (18) and an increased risk of hospitalization was reported in rituximab-treated patients (20).

The pathophysiology of SARS-CoV-2 is complex, with aggressive inflammatory responses implicated in the resulting damage to the airways; therefore, disease severity in patients is due to not only the viral infection but also to the host response which may cause a systemic involvement (22). In most individuals, recruited cells clear the infection in the lung, the immune response recedes, and the patient recovers. The adaptive immune response is necessary to eliminate the virus: this requires the presence of an appropriate genetic background in the host that activates specific antiviral immunity. In this phase of the infection, efficient immune responses are essential to avoid disease progression and virus propagation (23, 24).

However, in some patients, a dysfunctional immune response occurs, which triggers a cytokine storm that can lead to cytokine release syndrome (CRS) and that mediates widespread lung inflammation (22, 25). In this phase, strategies to suppress inflammation are required (24); immunosuppression might prevent the overly active immune response that drives tissue damage (26).

The initial responses against viruses are led mainly by T-lymphocytes and natural killer cells, and, to a lesser extent, by B-cells. This may be the reason why patients on anti-CD20 therapies cope generally well with viral infections. Anti-CD20 therapies have a relatively minor impact on T-cell counts and have not been associated with severe viral infections (27). These findings and the outcome observed in most of our patients suggest that the presence of circulating B cells is not necessarily required for recovery from COVID-19. Accordingly, we observed that four out of six tested cases showed the presence of IgG to SARS-COV2. Following these premises, many patients described in this case series had received the last infusion of ocrelizumab shortly before the onset of COVID-19 symptoms and, despite this, most of them developed a self-limiting disease that did not require hospitalization.

Patients with a higher MS-related disability in this series were likely to develop a more severe COVID-19 course, in line with the data reported from the French and Italian cohorts (18, 19).

Some of our patients reported prolonged and persistent symptoms even after several months of recovery. The time to symptoms resolution has been related to the severity of the acute illness and premorbid risk factors (28, 29). Some patients may also report prolonged neurological symptoms even without a premorbid neurological condition (30). Neurologic complications may be the consequences of the direct effects of the virus or the systemic response to the infection (31).

Moreover, given this possibility of long-term symptoms, many patients may require rehabilitation services, such as pulmonary and cardiac rehabilitation and physical therapy (32).

At the beginning of 2020, northern Italy was one of the first regions in Europe to be heavily hit by the first wave of the COVID-19 pandemic. The difficult challenges and uncertainties encountered by the healthcare system in the early phase of the COVID-19 pandemic might have contributed to the case fatality ratio observed in this series.

Registry-based studies suggested a higher risk of a more severe COVID-19 disease course in ocrelizumab-treated (18) and rituximab-treated MS patients (20). Recently, a report on COVID-19 in ocrelizumab-treated PwMS has been published by Roche clinical researchers. They concluded that COVID-19 severity in PwMS was in line with that of the general population and that case fatality rates were comparable with other MS cohorts (17). Potential limitations of this study are the lack of untreated MS controls matched to ocrelizumab-treated MS patients, the relatively low number of patients included, and the unavailability of serological data on the SARS-CoV2 antibody status for some of the patients.

In conclusion, this real-life case series highlights the variability of the course of COVID-19 in ocrelizumab-treated MS patients. The decision of treating a patient with ocrelizumab during the COVID-19 pandemic needs to be discussed between the patient and the treating neurologist, based on a benefit/risk assessment specific to the individual patient. A position paper was recently issued by expert clinicians regarding ocrelizumab use during the Covid-19 pandemic: considering that experiences coming from the first pandemic wave have not been brought to different risk stratification in terms of DMTs, these Authors suggest maintaining pre-pandemic criteria in the therapeutic choice (33); it is also suggested that these patients are immunized with seasonal-flu and COVID-19 vaccination (33).

The MS International Federation spread global advice to patients with MS, with measures to minimize the infection risk and implications associated with DMTs use (34). Recommendations from several scientific societies strongly urge to consider patients with MS patients treated with a disease-modifying treatment as a priority group for COVID-19 vaccination (35).

## Data Availability Statement

The raw data supporting the conclusions of this article will be made available by the authors, without undue reservation.

## Ethics Statement

Ethical review and approval was not required for the study on human participants in accordance with the local legislation and institutional requirements. The patients/participants provided their written informed consent to participate in this study. Written informed consent was obtained from the individual(s) for the publication of any potentially identifiable images or data included in this article.

## Author Contributions

SD, MV, and AA: data collection and paper writing. CB and VS: data collection. MC and PC: data collection, paper writing, and supervision. All authors contributed to the article and approved the submitted version.

## Conflict of Interest

The authors declare that the research was conducted in the absence of any commercial or financial relationships that could be construed as a potential conflict of interest. The handling editor declared a past co-authorship with one of the authors MC.

## Publisher's Note

All claims expressed in this article are solely those of the authors and do not necessarily represent those of their affiliated organizations, or those of the publisher, the editors and the reviewers. Any product that may be evaluated in this article, or claim that may be made by its manufacturer, is not guaranteed or endorsed by the publisher.
